# Low-Energy Extracorporeal Shock Wave Therapy Ameliorates Kidney Function in Diabetic Nephropathy

**DOI:** 10.1155/2019/8259645

**Published:** 2019-07-04

**Authors:** Chang-Chun Hsiao, Wei-Han Huang, Kuang-Hung Cheng, Chien-Te Lee

**Affiliations:** ^1^Graduate Institute of Clinical Medical Sciences, Chang Gung University, Taiwan; ^2^Center for Shockwave Medicine and Tissue Engineering, Kaohsiung Chang Gung Memorial Hospital, Taiwan; ^3^Institute of Biomedical Science, National Sun Yat-sen University, Taiwan; ^4^Division of Nephrology, Department of Internal Medicine, Kaohsiung Chang Gung Memorial Hospital and Chang-Gung University College of Medicine, Taiwan

## Abstract

**Background:**

Diabetic nephropathy is the most common cause of end-stage renal disease. Traditional therapy for diabetic nephropathy has focused on supportive treatment, and there is no significant effective therapy. We investigated the effect of low-energy extracorporeal shock wave therapy on a diabetic nephropathy rat model.

**Methods:**

Streptozotocin-induced diabetic nephropathy rats were treated with six sessions of low-energy extracorporeal shock wave therapy (weekly for six consecutive weeks) or left untreated. We assessed urinary creatinine and albumin, glomerular volume, renal fibrosis, podocyte number, renal inflammation, oxidative stress, and tissue repair markers (SDF-1 and VEGF) six weeks after the completion of treatment.

**Results:**

The six-week low-energy extracorporeal shock wave therapy regimen decreased urinary albumin excretion as well as reduced glomerular hypertrophy and renal fibrosis in the rat model of diabetic nephropathy. Moreover, low-energy extracorporeal shock wave therapy increased podocyte number in diabetic nephropathy rats. This was likely primarily attributed to the fact that low-energy extracorporeal shock wave therapy reduced renal inflammation and oxidative stress as well as increased tissue repair potency and cell proliferation.

**Conclusions:**

Low-energy extracorporeal shock wave therapy preserved kidney function in diabetic nephropathy. Low-energy extracorporeal shock wave therapy may serve as a novel noninvasive and effective treatment of diabetic nephropathy.

## 1. Introduction

Diabetic nephropathy (DN) is the most common cause of end-stage renal disease globally in association with the incidence rise in diabetes mellitus [1–3]. DN is a serious complication of diabetes mellitus [4, 5]. Clinical manifestations of DN are urine albumin excretion, glomerular hypertrophy, increased extracellular matrix production, and glomerular fibrosis [1, 6]. The pathogenesis of DN involves hyperglycemia [3], inflammation [3], oxidative stress [7], and angiotensin II [8]. Podocyte dysfunction or apoptosis not only plays an initial role in the development and progression of proteinuria but is also a key factor that drives glomerulosclerosis in the pathogenesis of DN [9].

The shock wave is a longitudinal acoustic wave, traveling at a speed of ultrasound waves in water through the body tissues [10]. Furthermore, the shock wave is a single pressure pulse with a short needle-like positive spike (1 *μ*s in duration and up to 100 MPa in amplitude) and followed by a tensile part of several microseconds at a lower amplitude [10, 11]. Low-energy extracorporeal shock wave therapy (ESWT) exerts its effects through other mechanisms, such as anti-inflammation [12, 13], inhibition of oxidative stress [14], improved regional blood flow [15], increased angiogenesis [16], and improved nerve regeneration [17, 18]. Given that low-energy ESWT exerts anti-inflammatory, inhibited oxidative stress, and improved nerve regeneration effects, low-energy ESWT may benefit the DN kidney. However, whether ESWT can alleviate DN disease is unknown. Therefore, we hypothesized that low-energy ESWT would enhance anti-inflammation, inhibit oxidative stress, and enhance antifibrosis and antiapoptotic effects to restore the renal function to normal level in the DN rat model by promoting podocyte regeneration.

## 2. Methods

### 2.1. Animals

Animal studies were approved by the Institutional Animal Care and Use Committee of Chang Gung Memorial Hospital (Approval No. 2016062205). Eighteen-week-old male Wistar rats weighing 400-470 g were used for the experiment, and these rats were purchased from BioLASCO (Taipei, Taiwan) and housed under controlled conditions of temperature (22–24°C) and illumination (12 : 12 h light-dark cycle, starting from 6:00 AM) and maintained with Purina Chow and tap water ad libitum. Thirty rats were randomized to the normal control group (*N* = 10), the DN group (*N* = 10), and the DN with ESWT group (*N* = 10).

### 2.2. Establishment of Diabetic Nephropathy Rat Model

Diabetes was induced in the overnight fasted rats by a one-time intraperitoneal injection of streptozotocin (STZ) (50 mg/kg, Sigma-Aldrich, St. Louis, MO) dissolved in citric acid buffer 40 mg/mL (pH 4.5, Sigma) [19–22]. However, the control group rats were injected only with citric acid buffer. The blood glucose level was examined 2 days after the STZ injection using a Contour glucose meter (Freestyle, Abbott Laboratories, Alameda, CA) via tail puncture. Animals exhibiting blood glucose levels more than 300 mg/dl were considered type 1 diabetic [20, 21]; otherwise, they were excluded from our study. Blood glucose in animals was maintained at 350 mg/dl by treatment with insulin (0.4 unit/rat, insulin glargine (rDNA origin) injection, Lantus®) [19, 20] maintained 12 weeks to establish the DN rat models successfully [23, 24].

### 2.3. Low-Energy Shock Wave Treatment

Six weeks after injection of STZ, the rats were anesthetized by inhalation of 2.0% isoflurane and placed in a supine position on a warming pad at 37°C for ESWT treatment. Using the ultrasound (Toshiba, Tokyo, Japan), the ultrasonic gel was applied to the abdomen after shaving their hair. Based on our previous study [21], the shock wave applicator (EvoTron R05, High Medical Technologies, Switzerland) with the probe was placed on the abdomen wall (kidneys) and a total of 200 shocks were delivered at an energy flow density of 0.13 mJ/mm^2^ at a frequency of 200 pulse per min [21]. The rats received ESWT treatment one time a week for 6 weeks (Supplemental Figure 1) [21, 25].

### 2.4. Measurement of Urine Creatinine and Albumin

Urine excretion of each animal was collected using a metabolic cage. We collected 16-hour urine samples from the normal, DN, and DN with ESWT groups. Urine albumin levels were determined by microalbuminuria ELISA kit (Abcam, Cambridge, UK), and the urinary creatinine was measured by using a microplate assay kit (Abcam, Cambridge, UK) according to the manufacturer's protocol.

### 2.5. Hematoxylin and Eosin Stain, Immunohistochemistry, and Immunofluorescence

Kidney tissues were fixed with 4% paraformaldehyde and embedded in paraffin. After deparaffinization and rehydration, the paraffin sections (3 *μ*m) were stained with hematoxylin and eosin according to the manufacturer's instructions. Mean glomerular volume (GV) was calculated according to the Weibel and Gomez formula [20, 26]. GV = (GA^1.5^ × 1.38)/1.01, mean glomerular profile area (GA) was calculated according to the ImageJ software (National Institutes of Health).

For immunohistochemistry, kidney tissues used were formalin-fixed and paraffin-embedded. The kidney tissue slides were washed three times with xylene for twelve~fifteen minutes per time, rehydrated in ethanol (100% twice for three minutes, 95% for three minutes), then rinsed with tap water for five minutes. Heat section in 10 mM citrate buffer at full power with a pressure cooker, then the kidney tissue slides were treated with 6% hydrogen peroxide for ten minutes at room temperature; peroxidase block (Novolink Polymer Detection System, Leica, Germany) removed endogenous peroxidase for ten minutes, and slides were washed in TBS for five minutes for three times; protein block was fifteen minutes, slides were washed in TBS for five minutes for three times, and primary antibody anti-collagen I (Abcam, Cambridge, UK), anti-TGF-*β* (Abcam, Cambridge, UK), anti-PCNA (Abcam, Cambridge, UK), anti-WT-1 (Abcam), anti-CD68 (Abcam), or anti-8-OHDG was added at room temperature for one hour; slides were washed in TBS for five minutes for three times, secondary antibody was added at room temperature for thirty minutes, slides were washed in TBS for five minutes for three times, and DAB reagent was added for colorimetric detection in few seconds and hematoxylin 20 seconds. Dehydrate slides/tissues and mount the slides. Slides were viewed under a Carl Zeiss Axioskop 2 plus microscope (Carl Zeiss, Germany). Image processing was completed using Image-Pro Plus software (Media Cybernetics).

For immunofluorescence, kidney cryosections (prepared in Tissue-Tek OCT compound, Sakura Finetek) were fixed with cold methanol/acetone (1 : 1) for 10 minutes at room temperature and immersed in 0.2% Triton X-100 for 10 minutes. After blocking with 10% horse serum in PBS for 1 hour, the slides were immunostained with primary antibodies CD206 (Abcam, Cambridge, UK), F4/80 (Santa Cruz), and anti-SDF-1 (Santa Cruz, Dallas, Texas). Primary Abs were typically used at 1 : 200 or 1 hour at room temperature or 4°C overnight. Specimens were subsequently incubated with suitable fluorescent secondary antibodies (Invitrogen, Carlsbad, CA) for 1 hour and mounted in fluorescent mounting medium (Vector, Burlingame, CA). Imaging was performed using an Olympus confocal microscope (Olympus FluoView 1000, Japan) and Nikon Eclipse E600 microscope equipped.

### 2.6. Terminal Deoxynucleotidyl Transferase dUTP Nick End Labeling (TUNEL)

Apoptotic cell death was determined by using TUNEL staining (Roche Diagnostics, Germany) according to the manufacturer's instructions.

### 2.7. Western Blotting

Kidneys were lysed into RIPA-buffer for 10 minutes on ice. Protein concentration was determined with bichloroacetic acid protein assay kit (Pierce, Rockford, IL). Equal amounts (30–50 *μ*g) of protein extracts were loaded and separated by SDS-PAGE using 7.5%, 10%, 12.5%, and 15% polyacrylamide gels by 100 V for 140 min and then transferred to the polyvinylidene fluoride membrane for 3 hours (Millipore, Bedford, MA). Membranes were blocked with 4% skimmed milk in TBS-0.5% Tween 20 for 1 hour and then incubated with primary antibody anti-*β*-actin (Sigma, USA), anti-CD68 (Abcam), anti-TGF-*β* (Abcam, UK), CD206 (Abcam, UK), anti-WT-1 (Abcam, UK), anti-caspase-3 (Cell Signaling, Danvers, MA), anti-collagen-I (Abcam, UK), anti-HO-1 (Abcam), and PCNA (Abcam, UK) for 4°C overnight. The membranes were washed using TBS-0.5% Tween 20 and incubated with secondary antibodies, peroxidase-conjugated anti-rabbit (1 : 40000, Sigma, USA) and anti-mouse (1 : 40000, Sigma, USA) for 1 hour at room temperature. The signals on the membrane were detected using Immobilon Western Chemiluminescent HRP Substrate Kit (GE, USA) and exposed to X-ray film (GE, USA) for autoradiogram.

### 2.8. Real-Time Quantitative RT-PCR Analyses of mRNA

Total RNA from the kidneys was isolated by using Trizol reagent (Invitrogen, Carlsbad, CA) according to the manufacturer's protocol. The TaqMan Reverse Transcription Kit (Applied Biosystems, Foster City, CA) and a Gene Amp by Bio-Rad My Cycler (Bio-Rad, Hercules, CA) were used to generate cDNA. Gene expression analysis was determined by quantitative real-time PCR using the SYBR Green Master Mix and a 7500 Real-time PCR System (Applied Biosystems, Foster City, CA). The expressions of mRNA were normalized to the expression level of GAPDH mRNA and are relative to the average of all ΔCt values in each sample using the cycle threshold Ct method.

### 2.9. Enzyme-Linked Immunosorbent Assay (ELISA) Analysis

Kidney expression of cytokines, such as IL-1*β*, IL-6, and IL-10, was determined using the Quantikine ELISA Kit in accordance with the protocol specified by the manufacturer (R&D Systems, Minneapolis, MN).

### 2.10. Animals and Low-Energy ESWT

**Figure 1:**
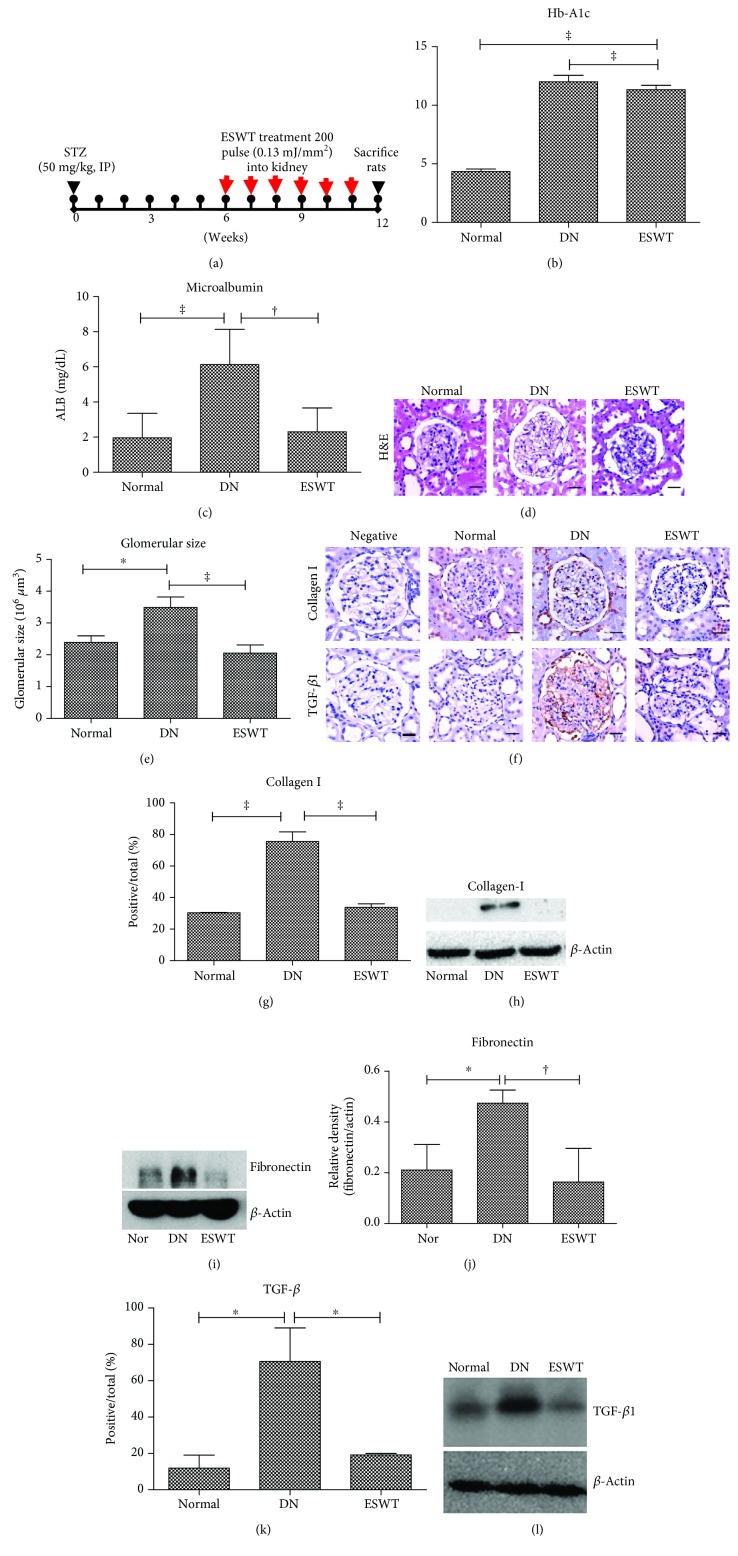
Low-energy ESWT improved renal function in DN. (a) Low-energy ESWT treatment protocol for DN rats. Arrows indicate that DN rats received ESWT (200 shocks, energy density of 0.13 mJ/mm^2^) for 6 weeks and then were killed at 12 weeks for study. (b) Blood hemoglobin A1c (HbA1c). Data are represented as mean ± SEM (*N* = 10); ^‡^ *P* < 0.005 versus the normal group. (c) Urine albumin (ALB). Data are represented as mean ± SEM (*N* = 10); ^‡^ *P* < 0.005, ^†^ *P* < 0.01. (d) Representative images of kidney tissue stained with hematoxylin and eosin (H&E); bar = 30 *μ*m. (e) Glomerular volume determined from H&E sections. (f, g, k) The renal fibrosis was determined by immunohistochemistry detection for type I collagen (Collage 1) and TGF-*β*1 in glomerular and quantification of immunohistochemistry staining by image analysis; bar = 30 *μ*m. (h, i, l, j) Western blot analysis Collage 1, fibronectin, and TGF-*β*1 expression in renal tissue and quantification of western blot by densitometric analysis. Normal group (Nor). (d–l) ^‡^ *P* < 0.005, ^†^ *P* < 0.01, and ^∗^ *P* < 0.05 (*N* = 6).

Thirty rats were randomized to the normal control group, the DN group, and the DN with ESWT group. Six weeks after STZ induction, low-energy ESWT was delivered weekly for 6 consecutive weeks (Figure 1(a)) [21, 25]. Blood glucose levels and body weight were measured weekly. Six weeks after the completion of ESWT, urine creatinine and albumin were assessed. Animals were then euthanized for ex vivo studies, including glomerular hypertrophy by glomerular volume, renal fibrosis by profibrotic markers transforming growth factor (TGF) *β*1, type I collagen and fibronectin, Wilms tumor 1 (WT-1) positive podocyte regeneration by proliferating cell nuclear antigen (PCNA), cell apoptosis markers TUNEL and Cleaved Caspase-3 (c-Caspase-3), CD68-positive macrophage infiltration and CD206-positive anti-inflammatory M2 macrophages, renal inflammatory mediators IL-6 and IL-1*β*, renal anti-inflammatory mediator IL-10, oxidative stress by 8-hydroxy-2′-deoxyguanosine (8-OHdG) and antioxidative stress marker heme oxygenase-1 (HO-1), tissue repair markers stromal cell derived factor 1 (SDF-1), and renal expression of VEGF.

### 2.11. Statistical Analysis

All data were expressed as the mean ± SEM from at least three independent experiments. The statistical significances of differences between two groups were determined using Student's *t*-test (*P* < 0.05). Quantitative real-time PCR data were calculated by 2^-ΔΔCt^ methods for mRNA quantification. All calculations were performed using the SPSS statistical software (version 13.0, SPSS, Chicago, IL, USA). Probability values of less than 0.05 were considered significant.

## 3. Results

### 3.1. Low-Energy ESWT Improved Renal Function in DN

Potential therapeutic effects of low-energy ESWT on DN rats were evaluated using a treatment protocol (Figure 1(a)) [21, 25]. The ESWT group showed no obvious differences from age-matched DN group rats in blood glucose (Supplemental Figure 2a). However, body weight in the DN group and the ESWT group was significantly decreased compared with the normal control group (Supplemental Figure 2b). The DN group had significantly increased blood hemoglobin A1c (HbA1c) compared with the normal control rats; however, ESWT did not reduce the elevated blood HbA1c level in the DN rats (Figure 1(b)). Albuminuria reflects renal dysfunction at the early stage of DN [27]. The DN group had significantly increased urinary albumin level compared with the normal control rats (Figure 1(c)). The ESWT had significantly reduced urinary albumin level compared with the DN group (Figure 1(c)). Glomerular hypertrophy, increased extracellular matrix production, and glomerular fibrosis are another two clinical manifestations of DN [1, 6]. Hematoxylin and eosin staining demonstrated that the DN group had significantly increased glomerular volume indicative of glomerular hypertrophy compared with the normal control rats (Figures 1(d) and 1(e)), whereas low-energy ESWT prevented glomerular hypertrophy in STZ-induced DN rats (Figures 1(d) and 1(e)). Immunohistochemistry staining and western blotting analyses showed that the DN group had significantly elevated extracellular matrix production (type I collagen and fibronectin) and TGF-*β*1 level in the kidneys compared with normal control rats; however, low-energy ESWT prevented renal fibrosis in STZ-induced DN rats (Figures 1(f)–1(l)). Low-energy ESWT improved renal function in DN.

### 3.2. Low-Energy ESWT Enhanced Podocyte Regeneration

**Figure 2:**
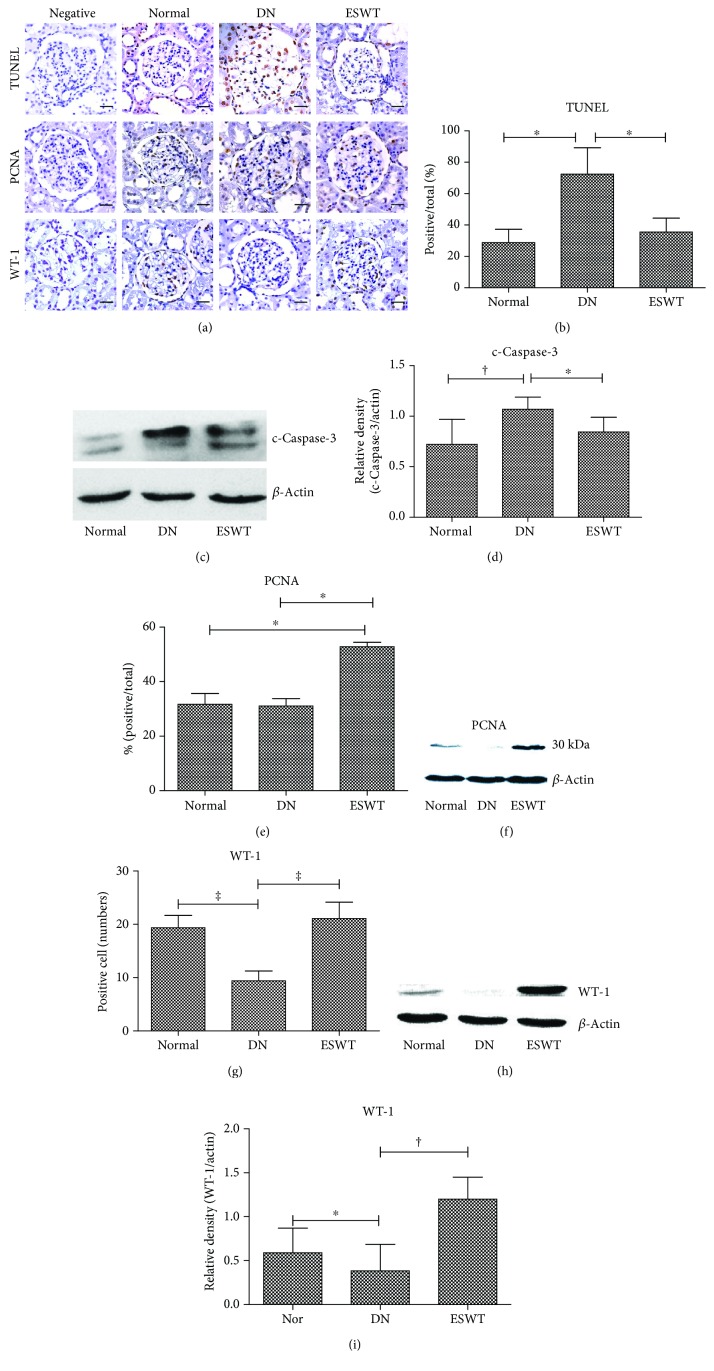
Low-energy ESWT enhanced podocyte regeneration. (a, b, e, g) Representative images of renal tissue immunohistochemistry stained with TUNEL indicating cell apoptosis, PCNA for evaluation of cell proliferation, and WT-1 indicating podocyte number and quantification of immunohistochemistry staining by image analysis; bar = 30 *μ*m. (c, f, h, d, i) Western blot analysis c-Caspase-3, PCNA, and WT-1 expression in renal tissue and quantification of western blot by densitometric analysis. Normal group (Nor). ^∗^ *P* < 0.05, ^‡^ *P* < 0.001, and ^†^ *P* < 0.01 (*N* = 6).

DN is associated with podocyte injury and loss [9]. Immunohistochemistry TUNEL staining and western blotting analyses revealed that the DN group had significantly increased cell apoptosis in the kidneys compared with normal control rats; however, low-energy ESWT ameliorated cell apoptosis in the DN kidneys (Figures 2(a)–2(d)). Moreover, ESWT had significantly increased cell proliferation in the kidneys compared with DN rats (Figures 2(a), 2(e), and 2(f)). The marker of podocyte is WT-1 [28]. The DN group had significantly reduced number of podocytes in the kidneys compared with normal control rats; however, ESWT had significantly increased number of podocytes in the kidneys compared with DN rats (Figures 2(a) and 2(g)–2(i)), suggesting that low-energy ESWT enhanced podocyte regeneration potency.

### 3.3. Low-Energy ESWT Prevented Diabetes-Induced Renal Inflammation

**Figure 3:**
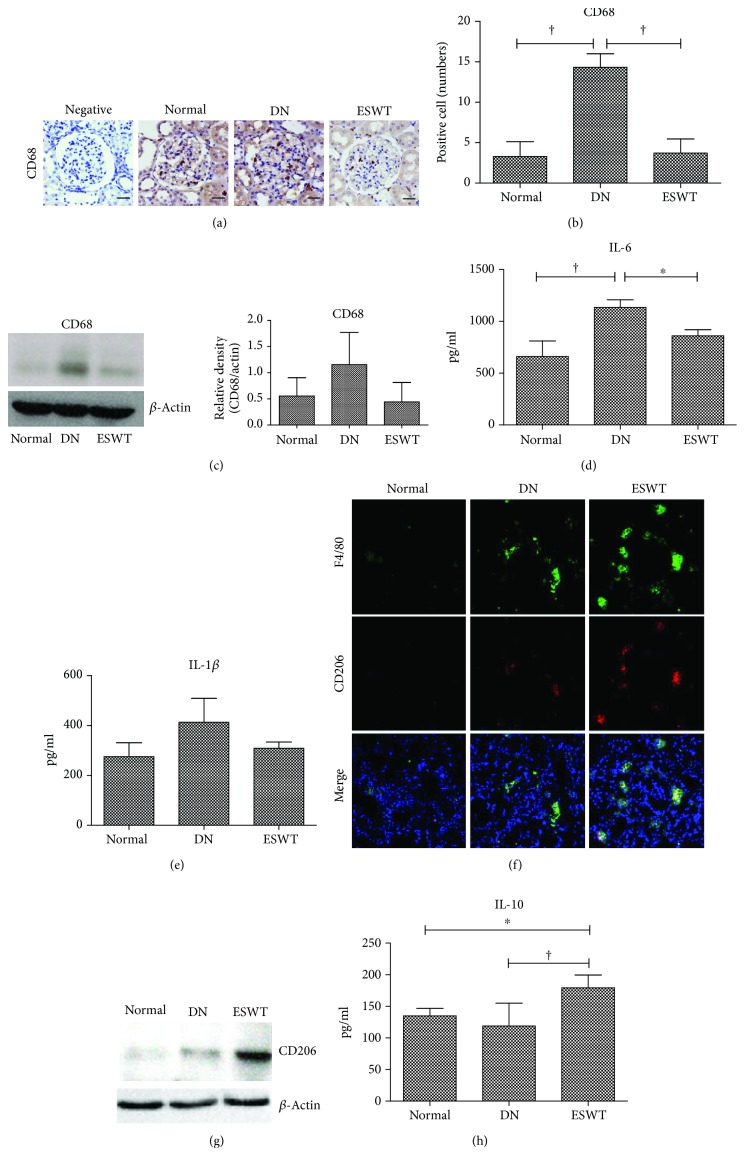
Low-energy ESWT prevented diabetes-induced renal inflammation. (a, b) Representative images of renal tissue immunohistochemistry stained with CD68 indicating macrophage infiltration and quantification of immunohistochemistry staining by image analysis; bar = 30 *μ*m. (c) Western blot analysis CD68 expression in renal tissue and quantification of western blot by densitometric analysis. (d, e) Renal expression of inflammatory mediators IL-6 and IL-1*β*. (f) Representative images of renal tissue immunofluorescence stained with F4/80 (green) and CD206 (red) indicating anti-inflammatory M2 macrophages. (g) Renal expression of anti-inflammatory M2 macrophage marker CD206. (h) Renal expression of anti-inflammatory mediator IL-10. ^†^ *P* < 0.01 and ^∗^ *P* < 0.05 (*N* = 6–10).

Inflammation is a crucial factor in the pathogenesis of DN [3]. CD68 staining and western blotting analysis revealed significantly increased macrophage infiltration in the DN kidney which was ameliorated by ESWT (Figures 3(a)–3(c)). The DN group exhibited high levels of IL-6 and IL-1*β* in the renal tissue, whereas ESWT significantly reduced the levels of these inflammatory mediators (Figures 3(d) and 3(e)). Moreover, ESWT had significantly increased anti-inflammatory M2 macrophages and anti-inflammatory mediator IL-10 in the kidneys compared with DN rats (Figures 3(f)–3(h)). Low-energy ESWT prevented diabetes-induced renal inflammation.

### 3.4. Low-Energy ESWT Alleviated Oxidative Stress and Enhanced Tissue Repair

**Figure 4:**
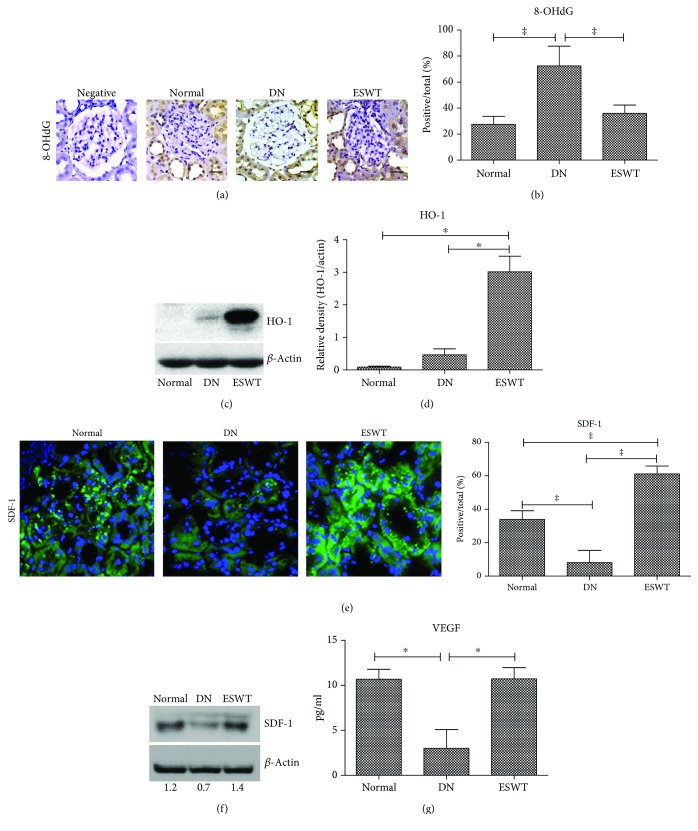
Low-energy ESWT alleviated oxidative stress and enhanced tissue repair. (a, b) Representative images of renal tissue immunohistochemistry stained with 8-OHdG indicating oxidative stress and quantification of immunohistochemistry staining by image analysis; bar = 30 *μ*m. (c, d) Renal expression of antioxidative stress marker HO-1. (e) Representative images of renal tissue immunofluorescence stained with SDF-1 (green) indicating tissue repair potency and quantification of immunofluorescence staining by image analysis. (f, g) Renal expression of tissue repair markers SDF-1and VEGF. ^‡^ *P* < 0.005, ^†^ *P* < 0.01, and ^∗^ *P* < 0.05 (*N* = 6–9).

Oxidative stress is a crucial factor in the pathogenesis of DN [7]. Immunohistochemistry 8-OHdG staining revealed significantly increased oxidative stress in the DN kidney which was ameliorated by ESWT (Figures 4(a) and 4(b)). Moreover, ESWT had significantly increased level of antioxidative stress compared with DN rats (Figures 4(c) and 4(d)). The DN group had significantly reduced levels of SDF-1 and VEGF in the kidneys compared with normal control rats, whereas ESWT had significantly increased levels of these tissue repair markers in the kidneys compared with DN rats (Figures 4(e)–4(g)), suggesting that low-energy ESWT enhanced tissue repair potency.

## 4. Discussion

This study demonstrated that low-energy ESWT decreased urinary albumin excretion as well as reduced glomerular hypertrophy and renal fibrosis in the rat model of DN. Moreover, low-energy ESWT increased podocyte number in DN rats. This was likely primarily attributed to the fact that low-energy ESWT reduced renal inflammation and oxidative stress as well as increased tissue repair potency and cell proliferation. We also found that ESWT can alleviate or prevent podocyte loss which may contribute to the preservation of kidney function. This study suggests that low-energy ESWT is a novel noninvasive and effective treatment of DN. “Low-Intensity Shock Wave Therapy in the Treatment of Diabetic Nephropathy: A Prospective Phase 1 Study” by Skov-Jeppesen et al. have results that did not demonstrate significant improvements of GFR and albuminuria [29]. The therapeutic effects were not as significant in the prospective phase 1 study by Skov-Jeppesen et al. as those in the present study, the possible causes are different therapeutic settings including energy levels, number of shots, and frequency of treatment (Table 1).

Inflammation is recognized as a major factor driving the pathogenesis of DN [3, 30]. This study shows that low-energy ESWT significantly decreased the proinflammatory molecules IL-6, IL-1*β*, and CD68-positive M1 macrophages but increased the anti-inflammatory mediators IL-10 and CD206-positive M2 macrophages, which may contribute to the preservation of renal function. Low-energy ESWT is also reported to enhance M2 macrophage infiltration in a rat model of acute myocardial infarction [31]. These results suggested that low-energy ESWT might inhibit the inflammation by polarizing M1 macrophages to M2 macrophages. Low-energy ESWT has an anti-inflammatory effect against various diseases [12, 13, 32, 33].

Low-energy ESWT generates mechanical forces and exerts biological effects [34]; after which, upregulation of tissue repair markers, including SDF-1 [32, 35] and VEGF [32, 35, 36], and reduction of oxidative stress [14] may thereby facilitate podocyte regeneration and tissue repair. Low-energy ESWT is also reported to be effective in ameliorating renal dysfunction in a rat model of acute kidney injury, through the stimulation of VEGF expression [37]. Interestingly, our previous study has also shown that low-energy ESWT can activate axonal regeneration [21]. Low-energy ESWT can improve nerve regeneration [17] and peripheral nerve regeneration [18]. The effects of repeated extracorporeal shock wave on kidney apoptosis of normal and diabetic rat were studied by Kira et al. and the results failed to show a significant beneficial impact on apoptosis [38]. However, low-energy ESWT ameliorated apoptosis of DN kidneys in the present study, the possible causes are different therapeutic settings including energy levels, number of shots, and frequency of treatment compared with Kira et al.'s study (Table 1).

**Figure 5:**
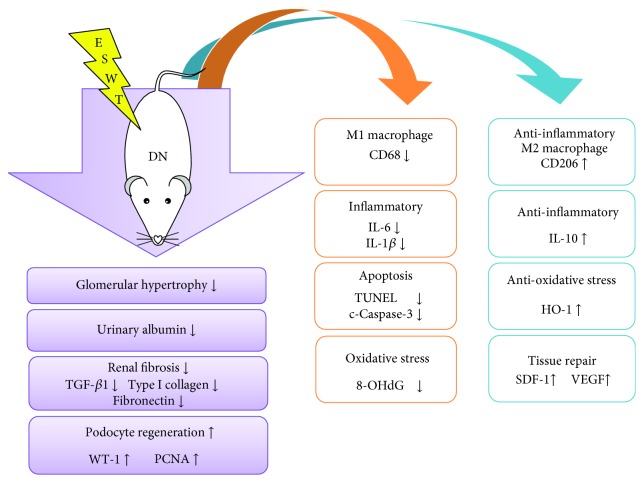
Proposed mechanisms underlying the therapeutic effects of low-energy ESWT on DN.

The underlying mechanisms in the therapeutic effect of ESWT against various diseases have been investigated, including inhibition of inflammation [12, 13] and oxidative stress [14] as well as enhancement of angiogenesis [16]. In this study, although extensive work has been done to elucidate the therapeutic effect of ESWT on DN, the exact underlying mechanisms remain unclear. The proposed mechanisms underlying the therapeutic effect of ESWT on DN based on our findings have been summarized (Figure 5).

## Figures and Tables

**Table 1:** Low-energy ESWT treatment protocol.

Group	Energy	Shock waves	Times	Equipment
Skov-Jeppesen^∗^	0.265 mJ/mm^2^	3000	Six times during a 3-week period	Modulith SLX-2^+^
Kira et al.^†^	14 kVA	2000	Two times during a 2-week period	Tripter X1^&^
Present study	0.13 mJ/mm^2^	200	Six times during a 6-week period	EvoTron R05^#^

^∗^ *Nephrol Dial Transplant* (2018) 1-8; ^+^Storz Medical AG, Switzerland; ^†^ *International Braz J Urol* (2008) 34: 91-96; ^&^Direx, Israel; ^#^High Medical Technologies, Switzerland.

## Data Availability

The data used to support the findings of this study are available from the corresponding author upon request.

## Supplementary Materials

Supplementary Materials Supplemental Figure 1: illustration of a low-energy extracorporeal shock wave therapy (ESWT) experiment set-up. (a) Using ultrasound probe and ultrasound system localizes for kidney position. (b) Ultrasound kidney image. (c) Low-energy ESWT treatment zone along the kidney. (d) Ultrasound system. (e) Low-energy extracorporeal shock wave system. Supplemental Figure 2: (a) blood glucose (mg/dl). (b) Body weight. Data are represented as mean ± SEM (*N* = 10); ^‡^ *P* < 0.005 versus the normal group.
