# Supplementary material for: Low-Energy Extracorporeal Shock Wave Therapy Ameliorates Kidney Function in Diabetic Nephropathy
Source: Oxid Med Cell Longev. 2019 Jul 4;2019:8259645. doi: 10.1155/2019/8259645 (PMC6637677; doi:10.1155/2019/8259645)

Supplemental Information

Low-Energy Extracorporeal Shock Wave Therapy Ameliorates Kidney Function in Diabetic Nephropathy

Chang-Chun Hsiao, Wei-Han Huang, Kuang-Hung Cheng, Chien-Te Lee

FIGURE LEGENDS

Supplemental Figure 1. Illustration of a low-energy extracorporeal shock wave therapy (ESWT) experiment set-up. (a) Using ultrasound probe and ultrasound system localizes for kidney position. (b) Ultrasound kidney image. (c) Low-energy ESWT treatment zone along the kidney. (d) ultrasound system. (e) Low-energy extracorporeal shock wave system.

Supplemental Figure 2. (a) Blood glucose (mg/dl). (b) Body weight. Data are represented as mean ± SEM (N = 10); $^{\ddagger}p<0.005$ versus normal group.

Supplemental Figure 1

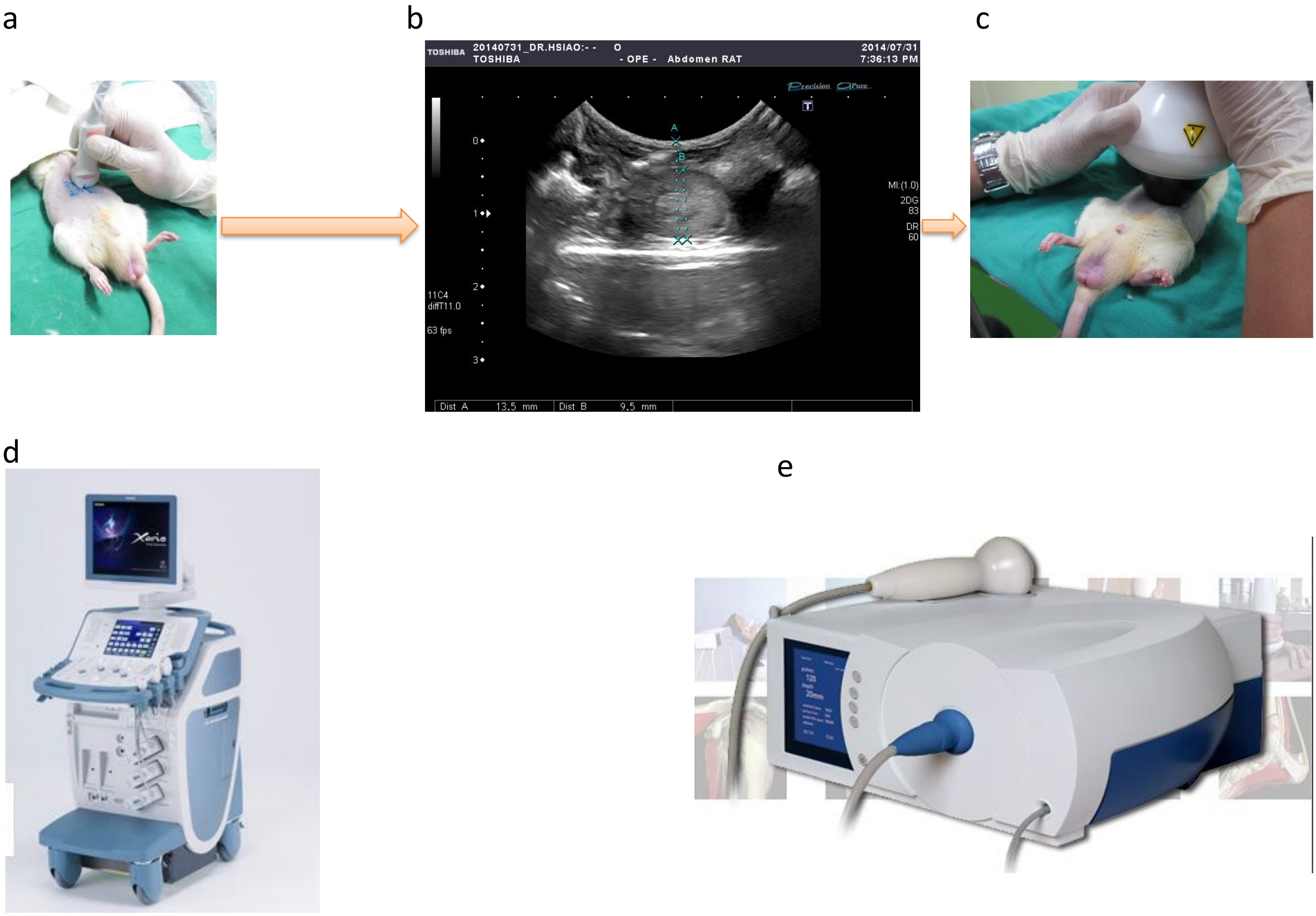

Supplemental Figure 2

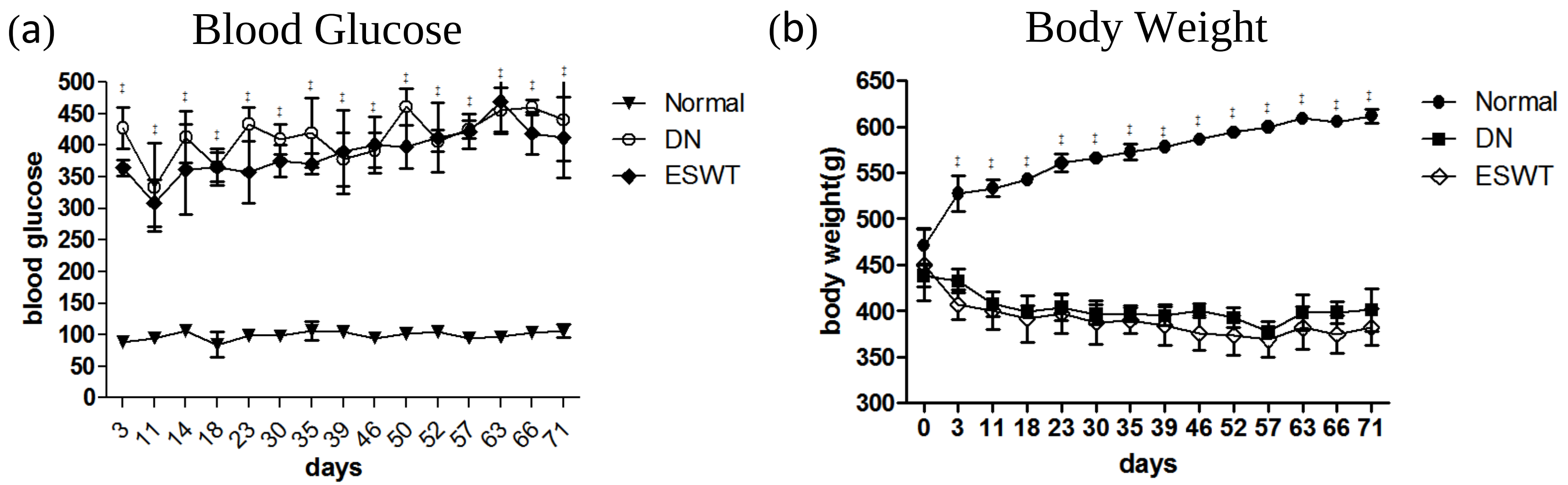

Supplement: Supplementary Materials — Supplemental Figure 1: illustration of a low-energy extracorporeal shock wave therapy (ESWT) experiment set-up. (a) Using ultrasound probe and ultrasound system localizes for kidney position. (b) Ultrasound kidney image. (c) Low-energy ESWT treatment zone along the kidney. (d) Ultrasound system. (e) Low-energy extracorporeal shock wave system. Supplemental Figure 2: (a) blood glucose (mg/dl). (b) Body weight. Data are represented as mean ± SEM (N = 10); ‡ P < 0.005 versus the normal group. [file 8259645.f1.pdf]
